# Supplementary material for: Comparative Mitogenome Analyses of Fifteen Ramshorn Snails and Insights into the Phylogeny of Planorbidae (Gastropoda: Hygrophila)
Source: Int J Mol Sci. 2024 Feb 14;25(4):2279. doi: 10.3390/ijms25042279 (PMC10889216; doi:10.3390/ijms25042279)
Supplement: Supplementary file 1 [file ijms-25-02279-s001.zip › ijms-2840374-supplementary.pdf]

Table S1. Characteristics of *Anisus vortex* mitogenome.

**Gene[1]**	**Location**	**Size (bp)**	**INC[2] (bp)**	**AT%**	**AT-skew**	**GC-skew**	**Start codon**	**Stop codon**
F	1-62	62	0	79.0%	0.061	0.385		
cox2	63-710	648	0	70.7%	-0.087	0.021	ATT	TAA
Y	711-748	38	0	68.4%	0.000	0.000		
G	749-807	59	0	78.0%	-0.130	-0.385		
H	801-863	63	-7	79.4%	-0.160	0.538		
Q	863-920	58	-1	62.1%	0.056	0.273		
L2	921-975	55	0	74.5%	-0.122	0.286		
atp8	974-1087	114	-2	80.7%	-0.043	-0.273	ATA	TAA
N	1,089-1,151	63	1	76.2%	0.125	0.333		
atp6	1,152-1,791	640	0	77.5%	-0.121	0.028	ATT	T
R	1,792-1,855	64	0	78.1%	-0.120	-0.143		
E	1,864-1,912	49	8	87.8%	0.023	0.000		
rrnS	1,913-2,610	698	0	74.4%	0.118	0.028		
M	2,611-2,674	64	0	73.4%	0.277	-0.176		
nad3	2,675-3,014	340	0	81.8%	-0.122	0.097	ATA	T
S2	3,016-3,069	54	1	79.6%	0.163	0.091		
S1	3,068-3,124	57	-2	73.7%	-0.143	0.067		
nad4	3,125-4,429	1305	0	76.7%	-0.203	0.007	ATA	TAA
T	4,441-4,501	61	11	78.7%	-0.042	0.077		
cox3	4,502-5,303	802	0	70.9%	-0.139	0.082	ATT	T
I	5,318-5,382	65	14	67.7%	-0.182	0.238		
nad2	5,399-6,304	906	16	78.4%	-0.172	0.194	ATT	TAA
K	6,264-6,329	66	-41	69.7%	-0.304	0.200		
cox1	6,324-7,856	1,533	-6	69.7%	-0.244	0.112	TTG	TAA
V	7,849-7,910	62	-8	77.4%	-0.125	0.143		
rrnL	7,911-8,897	987	0	79.5%	-0.045	0.129		
L1	8,898-8,958	61	0	73.8%	-0.244	0.500		
A	8,956-9,015	60	-3	78.3%	-0.021	0.385		
P	9,016-9,077	62	0	69.4%	-0.070	0.263		
nad6	9,078-9,524	447	0	81.9%	-0.301	0.185	ATA	TAA
nad5	9,517-11,154	1,638	-8	75.6%	-0.196	0.143	TTG	TAG
nad1	11,135-12,043	909	-20	74.9%	-0.189	0.149	ATG	TAA
nad4L	12,040-12,330	291	-4	80.1%	-0.202	0.172	ATA	TAA
W	12,329-12,390	62	-2	74.2%	0.000	-0.250		
C	12,391-12,450	60	0	83.3%	0.000	0.000		
cob	12,451-13,510	1,060	0	71.9%	-0.218	0.074	ATA	T
D	13,513-13,566	54	2	74.1%	-0.050	0.143		

[1] Genes underlined are on the minor strand; genes not underlined are on the major strand; tRNA genes are indicated with one-letter corresponding amino acids. [2] INC: intergenic nucleotides; positive values indicate gaps and negative values indicate overlapped nucleotides between adjacent genes.

Table S2 The best partitioning scheme selected by PartitionFinder for different datasets.

Dataset	Best model	Subset partitions
P123	F81+G+X	*atp8*_pos2
	GTR+G+X	*cox1*_pos1, *nad2*_pos2, *nad4*_pos2, *nad6*_pos2
	GTR+I+G+X	*atp6*_pos1, *atp6*_pos2, *cob*_pos1, *cox2*_pos1, *cox3*_pos1, *nad1*_pos1, *nad2*_pos1, *nad3*_pos1, *nad4*_pos1, *nad4L*_pos1, *nad5*_pos1, *nad5*_pos2, *nad6*_pos1
	GTR+I+X	*cox1*_pos2, *nad4L*_pos2
	HKY+G+X	*atp8*_pos3, *cob*_pos3, *cox2*_pos3, *nad1*_pos3, *nad3*_pos3
	HKY+I+G+X	*atp8*_pos1
	K81UF+G+X	*nad6*_pos3
	TIM+G+X	*atp6*_pos3, *cox3*_pos3
	TRN+G+X	*cox1*_pos3
	TRN+I+G+X	*nad2*_pos3, *nad5*_pos3, *nad4*_pos3, *nad4L*_pos3
	TVM+G+X	*nad3*_pos2
	TVM+I+G	*cox2*_pos2, *cox3*_pos2
	TVM+I+G+X	*cob*_pos2, *nad1*_pos2
P123R	F81+G+X	*atp8*_pos2
	GTR+G+X	*atp8*_pos1, *cox1*_pos1, *nad2*_pos2, *nad4*_pos2, *nad6*_pos2, *rrnS*
	GTR+I+G	*cox1*_pos2
	GTR+I+G+X	*atp6*_pos1, *cob*_pos1, *cox2*_pos1, *cox3*_pos1, *nad1*_pos1, *nad2*_pos1, *nad3*_pos1, *nad4*_pos1, *nad4L*_pos1, *nad5*_pos1, *nad6*_pos1
	GTR+I+X	*nad4L*_pos2
	HKY+G+X	*atp8*_pos3, *cob*_pos3, *cox2*_pos3, *nad1*_pos3, *nad3*_pos3
	K81UF+G+X	*nad6*_pos3
	TIM+G+X	*atp6*_pos3, *cox3*_pos3
	TRN+G+X	*cox1*_pos3
	TRN+I+G+X	*nad2*_pos3, *nad4*_pos3, *nad4L*_pos3, *nad5*_pos3
	TVM+G+X	*nad3*_pos2
	TVM+I+G+X	*atp6*_pos2, *cob*_pos2, *cox2*_pos2, *cox3*_pos2, *nad1*_pos2, *nad5*_pos2, *rrnL*
P12	F81+G+X	*atp8*_pos2
	GTR+G+X	*cox1*_pos1, *nad2*_pos2, *nad4*_pos2, *nad6*_pos2
	GTR+I+G+X	*atp6*_pos1, *atp6*_pos2, *cob*_pos1, *cox1*_pos2, *cox2*_pos1, *cox3*_pos1, *nad1*_pos1, *nad2*_pos1, *nad3*_pos1, *nad4*_pos1, *nad4L*_pos1, *nad5*_pos1, *nad5*_pos2, *nad6*_pos1
	GTR+I+X	*nad4L*_pos2
	HKY+I+G+X	*atp8*_pos1
	TVM+G+X	*nad3*_pos2
	TVM+I+G+X	*nad1*_pos2, *cob*_pos2, *cox2*_pos2, *cox3*_pos2
P12R	F81+G+X	*atp8*_pos2
	GTR+G+X	*atp8*_pos1, *cox1*_pos1, *nad2*_pos2, *nad4*_pos2, *nad6*_pos2, *rrnS*
	GTR+I+G	*cox1*_pos2
	GTR+I+G+X	*atp6*_pos1, *atp6*_pos2, *cob*_pos1, *cox2*_pos1, *cox3*_pos1, *nad1*_pos1, *nad2*_pos1, *nad3*_pos1, *nad4*_pos1, *nad4L*_pos1, *nad5*_pos1, *nad5*_pos2, *nad6*_pos1
	GTR+I+X	*nad4L*_pos2
	TVM+G+X	*nad3*_pos2
	TVM+I+G+X	*cob*_pos2, *cox2*_pos2, *cox3*_pos2, *nad1*_pos2, *rrnL*
